# Histone-based liquid biopsy discriminates between myelodysplastic syndrome and solid malignancies

**DOI:** 10.1186/s13148-025-01995-w

**Published:** 2025-11-21

**Authors:** Desislava K. Tsoneva, Diana Buzova, Salvatore Daniele Bianco, Ilina Micheva, Merlin Efraim, Nikol Daskalova, Igor Resnick, Maria Teneva, Trifon Chervenkov, Nikolay Vladimirov Conev, Rostislav Manev, Dragomir Svetozarov Stoyanov, Jan Cerveny, Tommaso Mazza, Manlio Vinciguerra

**Affiliations:** 1 Department of Medical Genetics, Medical University of Varna, Varna, Bulgaria; 2 Department of Stem Cell Biology and Transplantology, Research Institute of the Medical University of Varna, Varna, Bulgaria; 3 Department of Adaptive Biotechnologies, Global Change Research Institute CAS, Brno, Czech Republic; 4 Bioinformatics Laboratory, Fondazione IRCCS Casa Sollievo della Sofferenza, S. Giovanni Rotondo, Italy; 5 Clinic of Hematology, St. Marina University Hospital, Medical University of Varna, Varna, Bulgaria; 6 Laboratory of Clinical Immunology, St. Marina University Hospital, Varna, Bulgaria; 7 Clinic of Medical Oncology, St. Marina University Hospital, Medical University of Varna, Varna, Bulgaria; 8 Computational Biology and Bioinformatics Unit, Fondazione Policlinico Universitario Agostino Gemelli IRCCS, Rome, Italy

**Keywords:** Solid tumor malignancies, MDS, Liquid biopsy, Histone, Nucleosomes

## Abstract

**Background:**

Cancers can be hematological or solid, sharing many hallmarks, although their clinical behaviors are distinct. Identifying biomarkers that differentiate hematological from non-hematological malignancies could aid differential diagnosis by providing the basis for developing point-of-care diagnostic devices. In this respect, complex histone populations are secreted and detectable in biological fluids in various disease settings. To our knowledge, studies analyzing the circulating histone profile complexity by comparing healthy individuals, patients with hematological malignancies, and solid cancer patients are currently lacking.

**Results:**

We assessed the plasma histone signature of healthy subjects (*n* = 30), and of patients with myelodysplastic syndrome (MDS, *n* = 43), colorectal cancer (CRC, *n* = 39), lung cancer (non-small cell lung cancer [NSCLC, *n* = 15]), small cell lung cancer [SCLC, *n* = 4]), or breast cancer [BC, *n* = 16]). Principal component analysis (PCA) demonstrated the segregation of circulating histones and histone complexes between oncological and healthy patients. Individual histones (H2A, H2B, H3, H4, macroH2A1.1, and macroH2A1.2), histone dimers and nucleosomes were assayed by ImageStream(X)-advanced flow cytometry. We found general increases in circulating histone abundance in the blood of cancer patients versus healthy controls. MDS and solid cancers could be discriminated among themselves for an increased abundance of histones H2A and macroH2A1.2 (*p* < 0.01), and a decreased abundance of H2A/H2B/H3/H4 and H3/H4 histone complexes (*p* < 0.01). Moreover, macroH2A1.2 and H2A/H2B/H3/H4 levels negatively or positively correlated with age in healthy subjects versus MDS patients, respectively.

**Conclusions:**

Overall, we identified circulating histone signatures able to discriminate between solid and MDS, using a rapid and non-invasive imaging technology, which may improve patient diagnosis.

**Supplementary Information:**

The online version contains supplementary material available at 10.1186/s13148-025-01995-w.

## Background

Cancer is the main cause of premature death [1], surpassing cardiovascular diseases as the main cause of mortality in high-income countries [2, 3]. Solid tumors are neoplastic growths forming a three-dimensional tissue mass and arising from non-hematopoietic tissues. In contrast, hematologic malignancies originate in blood-forming tissues and are typically characterized by the widespread presence of abnormal (often blast) cells in the circulating blood. Among hematological malignancies, myelodysplastic syndromes (MDSs) are a group of clonal bone marrow neoplasms characterized by morphologically abnormal and ineffective hematopoiesis, with a progression to acute myeloid leukemia (AML), occurring in ~ 30% of patients [4–7]. MDS incidence increases progressively with advancing age, especially in individuals over 70 [8]. Accordingly, although MDS is relatively rare (~ 4.0 cases per 100.000 persons), it is a life-threatening condition. Its global burden doubled in the last three decades, primarily due to population growth and aging, and it is projected to grow significantly [9]. Of the non-hematological malignancies, the three most commonly developing solid cancers are colon, lung, and breast cancer. Together, they account for more than one-third of all cancers and cancer-related mortality [10], representing a substantial global health burden with a cumulative ~ 3.5 million people succumbing every year to these conditions [10]. Despite the advances in therapeutic strategies, the increasing incidence of MDS and common solid malignancies indicates the need to enhance the focus on prevention approaches.

The growing understanding of the tumor molecular signature and the incorporation of novel approaches have drastically improved the diagnostic accuracy [11, 12]. However, in many cases, access to the tumor for biopsy is substantially limited due to its anatomical location or small size, making biopsy either unfeasible, unsafe, or prone to failure. Regarding minimally invasive biomarkers’ evaluation, individual ones are widely used but may lack specificity and/or sensitivity in the early disease stages [13–16], rendering composite serum biomarker signatures a more promising diagnostic approach [17–19]. In this context, the development and evaluation of additional comprehensive diagnostic approaches remain an important objective. Moreover, there is obvious interest in identifying and offering biomarkers that can serve as a basis for the development of a point-of-care diagnostic device testing, based on fast, cost-effective, and on-site biosensor technologies [20, 21]. In this respect, liquid biopsies (LB) are transforming our approach to tumor early detection, screening, profiling, and determining therapeutic response by the isolation of heterogeneous tumor-derived or tumor-associated material from biological fluids in a non-invasive or minimally invasive manner, allowing for gentle repeated sampling, and consequently, monitoring the therapeutic response or presence of minimal residual disease [22, 23]. LB include analysis on the level of the genome, transcriptome, and proteome by the assessment of circulating tumor cells (CTCs), cell-free DNA (cfDNA), extracellular vesicles (EVs), and different types of RNAs such as miRNAs [24]. At present, evaluation of circulating histones, nucleosomes, and histone modifications can also meet the requirements of the LB and thus be considered as such [25, 26]. There are specific advantages characterizing circulating free histones as a biomarker class. For example, cfDNA is currently the best-established surrogate diagnostic and prognostic marker in cancer to date. It enables the detection of circulating tumor DNA (ctDNA) and key cancer-associated genetic mutations, facilitating treatment personalization, identification of drug resistance mechanisms, disease monitoring, and prognosis assessment [27]. CfDNA is widely used in clinical trials; however, it also presents substantial technical challenges, including a short half-life of approximately 60 min, which may affect reproducibility [28, 29]. Circulating histones/nucleosomes are instead stable for several days, and are emerging as potential biomarkers for cancer detection, monitoring, and prognosis both in hematological and solid malignancies [30, 31]. Current studies are mostly focused on whole/entire nucleosomes (histone octamers—classically composed of two copies of each of the histone proteins H2A, H2B, H3, and H4). Furthermore, most studies in the field rely on enzyme-linked immunosorbent assay (ELISA) assays, which offer limited multiplexing capabilities. We have recently discovered that a complex population made of individual histones, histone dimers, and histone variants is secreted and detectable in the blood and other biological fluids in various disease settings [25, 32–37]. Among the 19 histone variants described in humans, macroH2A1 is the biggest and most abundant known histone, and we and others have implicated it in the pathogenesis of both solid and blood-borne malignancies [38–47]. To our knowledge, studies analyzing this complexity and comparing the levels of individual histones and histone complexes between healthy individuals, patients with hematological malignancies, and solid cancer patients are currently lacking. In this study, we assessed the plasma histone signature of MDS patients and patients diagnosed with CRC, lung cancer (non-small cell lung cancer [NSCLC] or small cell lung cancer [SCLC]), or breast cancer (BC), as potential diagnostic biomarkers in oncology research and clinical practice, using imaging flow cytometry.

## Methods

### Patients and biofluids

The collection of human specimens was approved by the local Ethics Committee of the Medical University of Varna, in accordance with the Declaration of Helsinki. All participants provided written informed consent before enrollment in the study. Blood samples were collected at the time of diagnosis, before the initiation of any specific treatment. We followed the standard and robust blood sampling/storage standard operation procedure (SOP) of the UK BioBank [48]. Blood was collected in K2EDTA-coated collection tubes and centrifuged at 3000 *g* for 20 min at 4 °C. The plasma samples were stored at − 80 °C. A total of 147 patients were included in the study. 54 oncological patients (22 CRC, 15 NSCLC, 4 SCLC, and 13 breast cancer) were recruited by the Department of Medical Oncology at the University Hospital “St. Marina” in Varna, Bulgaria. Computed tomography (CT) and tissue biopsy were used to confirm the diagnosis, location, and tissue of origin. A total of 20 plasma samples (17 CRC and 3 breast cancer) were acquired from Audubon Bioscience (TX, USA). 43 MDS patients were recruited by the Department of Hematology at the University Hospital “St. Marina” in Varna, Bulgaria. MDS diagnosis was confirmed by clinical presentation, abnormal hematopoiesis, presence of < 20% blasts on bone marrow examination, and characterization of cytogenetic and molecular abnormalities. Control samples (*n* = 30) were obtained from Proteogenex (CA, USA). All samples from healthy individuals were confirmed negative for HIV, HBV, HCV, and syphilis. All acquired samples were collected in K2 EDTA-coated collection tubes. All patients in the study are of Caucasian ethnicity. The following parameters were recorded: sex (referring to a set of biological and physiological characteristics that are associated with features such as chromosomal genotype, gamete production, hormonal levels, internal and external anatomy, age, stage of the disease, size/extent of tumor (T), number of cancer-affected lymph nodes (N), and presence of distant metastasis (M).

### ImageStreamX-based detection of histones and histone complexes in plasma of healthy individuals and cancer patients

To measure the levels of histones and histone complexes in plasma, we applied our previously described ImageStream-based multichannel detection approach [37]. In short, 25 μl plasma samples were incubated with primary antibodies against H2A, macroH2A1.1, macroH2A1.2, H2B, H3, and H4 in a ratio of 1:50 overnight at 4 °C. Due to the available 4 fluorescent channels on ImageStream, the detection of the above-mentioned 6 histone species was conducted by immunostaining each plasma sample with 3 different sets of primary antibodies (Table 1). The next day, the samples were incubated for 2 h at room temperature with the respective secondary antibodies (Table 2). For each sample immunostaining, 20,000 objects were collected with the following laser configurations: (1) excitation laser-488 nm at 5 mW for Alexa Fluor® 488; (2) excitation laser-561 nm at 20mW for Alexa Fluor® 555 and Alexa Fluor® 594; (3) excitation laser-642 nm at 5mW for Alexa Fluor® 647. Brightfield images were collected in channel one, while fluorescent images were collected in channels 2–5.

**Table 1:** Combination of primary antibodies used for the immunostaining of plasma samples

Combination number	H2A variant; antibody	H2B, H3, H4; antibodies
I	Canonical H2A; Abcam, Ab18255; polyclonal	H2B; BioLegend, 606,302; monoclonal H3; LS Bio, LS-B6334-125; polyclonal H4; Abcam, Ab31830; monoclonal
II	macroH2A1.1; Cell Signaling Technology, 12455S; monoclonal	
III	macroH2A1.2; Cell Signaling Technology, 4827S; polyclonal

**Table 2:** Combination of secondary antibodies used for the immunostaining of plasma samples from healthy individuals and patients with CRC, SCLC, NSCLC, or breast cancer

Combination number	Antibodies
I	Goat anti-rabbit IgG H&L-Alexa Fluor® 488; Thermo Fisher Scientific, A-11008; Goat Anti-Rat IgG H&L—AlexaFluor® 594; Abcam, ab150160 Donkey Anti-Sheep IgG H&L—Alexa Fluor® 555; Abcam, ab150178
II	
III	Goat anti-mouse IgG H&L-Alexa Fluor® 647; Thermo Fisher Scientific, A-21235

To segregate the histone particles from all detected objects and identify specific subpopulations, the following gating strategies were applied: (1) selection of focused objects (Gradient RMS_Brightfield vs. Normalized frequency); (2) exclusion of aggregates (Raw min pixel_Brightfield vs. Modulation_Object_Brightfield), and (3) identification of objects with fluorescence. The levels of plasma histones and histone complexes are measured as a percentage of the gated object population.

### Principal component analysis (PCA)

PCA was performed in RStudio (version 4.4.3) on the averaged and log-transformed abundance of histone/histone complexes. The data were scaled before the analysis. PCA was performed by prcomp, and plots were generated using the ggplot2 library. Abundance scores with a value of 0 across all patients were omitted from the data. Similarly, we applied a cutoff of 0.8 correlation coefficient to identify strongly correlated variables, which were subsequently excluded from the analysis. Silhouette scores [49] and the Calinski–Harabasz [50] indexes were used as quantitative assessment of clustering separation. These metrics were computed across an increasing number of principal components (from 1 up to 9), and for each case, we performed 1000 permutation tests to generate a random baseline distribution.

### Statistical analyses

Statistical analyses on the abundance of histones and histone complexes were conducted in Python by using the SciPy library (version 1.11.3), GraphPad Prism (version 10.2.3), and *RStudio (version 4.4.3)*. Detection of histones H2B, H3, and H4 occurs in all three sets of antibodies. To account for the potential batch effect, the measured abundance of all histones and histone complexes was averaged and log-transformed. Pairwise Kruskal–Wallis test was applied to compare the levels of plasma histones and histone complexes between groups, followed by a post-hoc test, when required. Pairwise Wilcoxon rank-sum test was applied when two populations were compared, followed by a post-hoc test, when required. Bonferroni–Dunn method was applied to correct for multiple testing. Boxplots and scatter plots were created using Matplotlib (version 3.8.1), GraphPad Prism (version 10.2.3), and *RStudio (version 4.4.3)*. To correlate the levels of histone and histone complexes with the clinical parameters, we used nonparametric Spearman correlation.

## Results

### Characteristics of patient groups

A total of 30 healthy controls, 43 MDS patients, 39 CRC patients, 4 SCLC patients, 15 NSCLC patients, and 16 BC patients were enrolled in this cohort study. The detailed baseline characteristics of our study population are displayed in Table 3 (healthy controls), Table 4 (MDS), and Table 5 (CRC, SCLC, NSCLC, and BC). The median age of healthy controls was 62 years, and 60% of them were male. Demographic and clinical–pathological characteristics, including cytogenetics and associated risks and classification of MDS patients, are reported in Table 4. Their median age was 72 years old, and approximately 2/3 of them were males. MDS is more common in the elderly, and healthy controls available in our repository were on average slightly younger. At the time of sampling, all patients were anemic, with hemoglobin levels ≤ 110 g/L (reference range: 120–170 g/L). Among the 43 patients evaluated, 22 were thrombocytopenic, while 4 exhibited thrombocytosis (platelet count, PLT; reference range: 150–450 × 10^9^/L). Neutropenia, defined by an absolute neutrophil count (ANC) below the reference range of 2.5–7 × 10^9^/L, was observed in 29 patients. A majority of patients (*n* = 40) had a mild-to-moderate elevation of lactate dehydrogenase (LDH) levels (> 250 U/L). Erythropoietin (EPO) levels were abnormally elevated in all patients tested, ranging from 36 to > 750 mU/mL. Serum ferritin was elevated in 30 out of 37 assessed patients, with values ranging from 365 to 3172 μg/L, and was not clearly correlated with transfusion history. Bone marrow trephine biopsy revealed hypercellularity in 30 of 41 patients and hypocellularity in five. All patients showed marked dysplastic changes involving one (*n* = 5), two (*n* = 9), or all three (*n* = 27) hematopoietic lineages.

**Table 3:** Characteristics of study participants. Healthy controls

*Parameters*	Healthy individuals *n* = 30
Sex, *n* (Male/Females)	18/12
Age (years) median (IQ range)	61.5 (54.25–71.25)
BMI (kg/m^2^) median (IQ range)	26.02 (24.2–27.95)

**Table 4:** Characteristics of study participants. MDS patients

*Variable*	*MDS*
*n*, number of patients	43
Sex, *n* (Males/Females)	30/13
Age (years), median (IQ range)	72 (41–88)
Hb (g/L), median (IQ range)	77 (28–110)
WBC (× 10^9^/L), median (IQ range)	3.68 (1.15–10.3)
Plt (× 10^9^/L), median (IQ range)	162 (9–859)
ALC (× 10^9^/L), median (IQ range)	1.33 (0.31–3.55)
ANC (× 10^9^/L), median (IQ range)	1.84 (0.11–7.79)
LDH (U/L), median (IQ range)	357 (187–1977)
B2-MG (mg/L), median (IQ range)	2.8 (1.83–8)
Epo (mU/ml), median (IQ range)	242 (24.5–750)
Ferritin (µg/L), median (IQ range)	837 (11.31–3172)
Cellularity, *n*	Hyper, *n* = 30
	Hypo, *n* = 5
	Normo, *n* = 6
	Dry tap, *n* = 1
	Unknown, *n* = 1
N of dysplasia, *n*	1, *n* = 5
	2, *n* = 9
	3, *n* = 27
	Unknown, *n* = 2
BM blast (%), median (IQ range)	3 (1–25)
Cytogenetics, *n*	46,XY (*n* = 16)
	41–42, XX, del(5)(q12;q33),-C,-D,-D,-E,-19,-22, + mar (*n* = 1)
	46, XY, del(22)(q11.2) (*n* = 1)
	46, XY,del(20)(q11.2) (*n* = 1)
	46,XX,t(8;21) (*n* = 1)
	46,XX (*n* = 3)
	47,XY, + 8 (*n* = 2)
	46,XX, del5q (*n* = 5)
	46,XY, del16q (*n* = 1)
	46,XY,del(20)(q12), del5(q31), del7(q31), + 8 (*n* = 1)
	45,XY, del21 (*n* = 1)
	46,XY, del11q (*n* = 1)
	46,XY, del16q (*n* = 1)
	46,XX, -C, + mar (*n* = 1)
	46,XX, del5q, -19, -22 (*n* = 1)
	46,XY, del5 (*n* = 1)
	47,XX, + 8 (*n* = 1)
	Unknown (*n* = 4)
Cytogenetic risk, *n*	Very poor, *n* = 2
	Good, *n* = 25
	Intermediate, *n* = 9
	Very good, *n* = 1
	High, *n* = 2
	Unknown, *n* = 4
BM fibrosis, *n*	Gr2, *n* = 5
	Gr1, *n* = 11
	Gr1-2, *n* = 2
	Unknown, *n* = 25
WHO 2016 classification, *n*	MDS-MLD, *n* = 23
	MDS-RS, *n* = 3
	RARS, *n* = 1
	RAEB1, *n* = 2
	RAEB-T, *n* = 1
	MDS-5q, *n* = 4
	sMDS, *n* = 1
	RAEB2, *n* = 8
IPSS-R, *n*	Very low, *n* = 1
	Low, *n* = 15
	Intermediate, *n* = 14
	High, *n* = 10
	Very high, *n* = 3

**Table 5:** Characteristics of study participants. Patients with solid tumors: colorectal carcinoma (CRC), small cell lung cancer (SCLC), non-small cell lung cancer (NSCLC), or breast cancer

Parameters	CRC	SCLC	NSCLC	Breast
	*n* = 39	*n* = 4	*n* = 15	*n* = 16
Sex (M/F)	21/18	2/2	11/4	0/16
Age (years) median (IQ range)	69 (60.5–73)	61.5 (58.5–66.5)	68 (59.5–72)	66.5 (50.8–71.8)
Stage (III/IV)	23/16	0/4	0/15	12/4
T (1/2/3/4)	0/6/26/7	0/0/3/1	1/1/3/10	2/8/1/5
N(0/1/2/3/unknown)	4/16/17/0/2	1/1/2/0	3/4/3/5	1/7/7/1
M (0/1)	23/16	0/4	0/15	12/4

Demographic and cancer aggressiveness characteristics of patients affected by one of four types of solid cancers (CRC, SCLC, NSCLC, and BC) are reported in Table 5. Patients with solid cancers had on average similar ages (range 61–66) and were all advanced at stage III or IV of the disease: in particular, all SCLC and NSCLC patients, 41% of CRC patients, and 25% of BC patients were diagnosed with stage IV, thereby with metastatic (M) cancer. The tumor size (T) was variable, but 77% of patients with solid cancer (57/74) in our cohort displayed lymph node invasion (*n* = 2 or 3) (Table 5).

### Circulating histone signature in MDS versus solid cancer patients

**Fig. 1:**
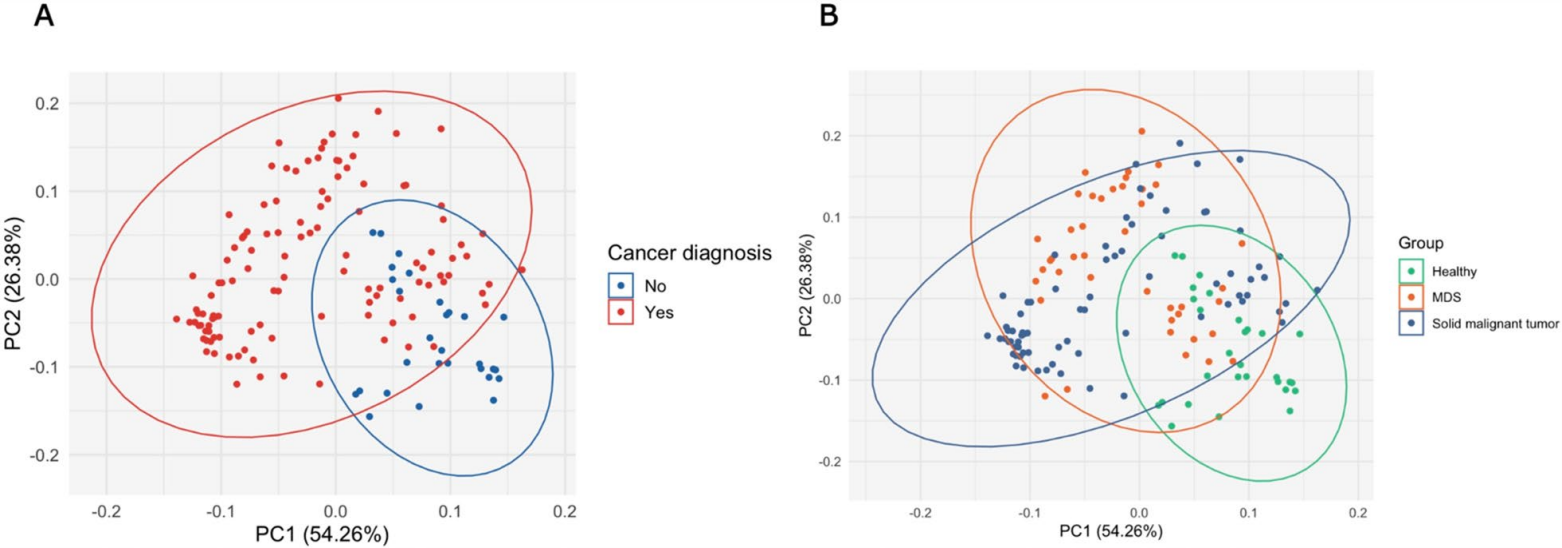
Principal component analysis (PCA) plot of the plasma histone profile in **A** healthy people and patients with malignancies; **B** healthy people and patients with malignancies of either hematological or solid tumor malignancies

To our knowledge, a comprehensive comparative analysis of circulating histone expression levels in plasma of hematological malignancies *versus* solid malignancies *versus* healthy controls has never been performed. To get a first glance at the diagnostic value of the different histone signatures between the study cohorts, we applied a PCA. We first compared the control population to patients with malignant disease, regardless of the origin (hematological or solid tumor) (Fig. 1A). The two populations are visually separated on the PCA plot, with the two principal components (PCs) explaining 80% of the variance (Fig. 1A). We then subdivided the cohort with malignant diseases into patients with MDS or solid malignant tumors (Fig. 1B). While some overlap between the different populations can be observed, samples from patients with MDS, solid tumor malignancies, and healthy controls show different segregation patterns on the PC plot (Fig. 1B). While the overlap between solid cancers and MDS seems substantial, 8 histone species were used in the analysis (H2A, H3, H4, macroH2A1.1, macroH2A1.2, H3/H4, H2A/H2B/H3/H4, macroH2A1.1/H2B/H3/H4). Therefore, small but significant differences in plasma levels of specific histone species could be obscured by the influence of other histone variables in the analysis. In order to quantitatively assess the separation between MDS and solid cancer in the PC analysis space (Supplementary Fig. 1A), we evaluated both the silhouette score (Supplementary Fig. 1B) and the Calinski–Harabasz index (Supplementary Fig. 1C). The results indicate that with two or more principal components, the separation between MDS and solid cancer becomes significantly greater than expected under random conditions. Specifically, when evaluating the observed metrics against the null hypothesis of random separation, the cumulative distribution function (CDF) approaches ~ 1 for component numbers greater than one, thereby confirming the statistical robustness of the observed group separation (Supplementary Fig. 1B, C).

**Fig. 2:**
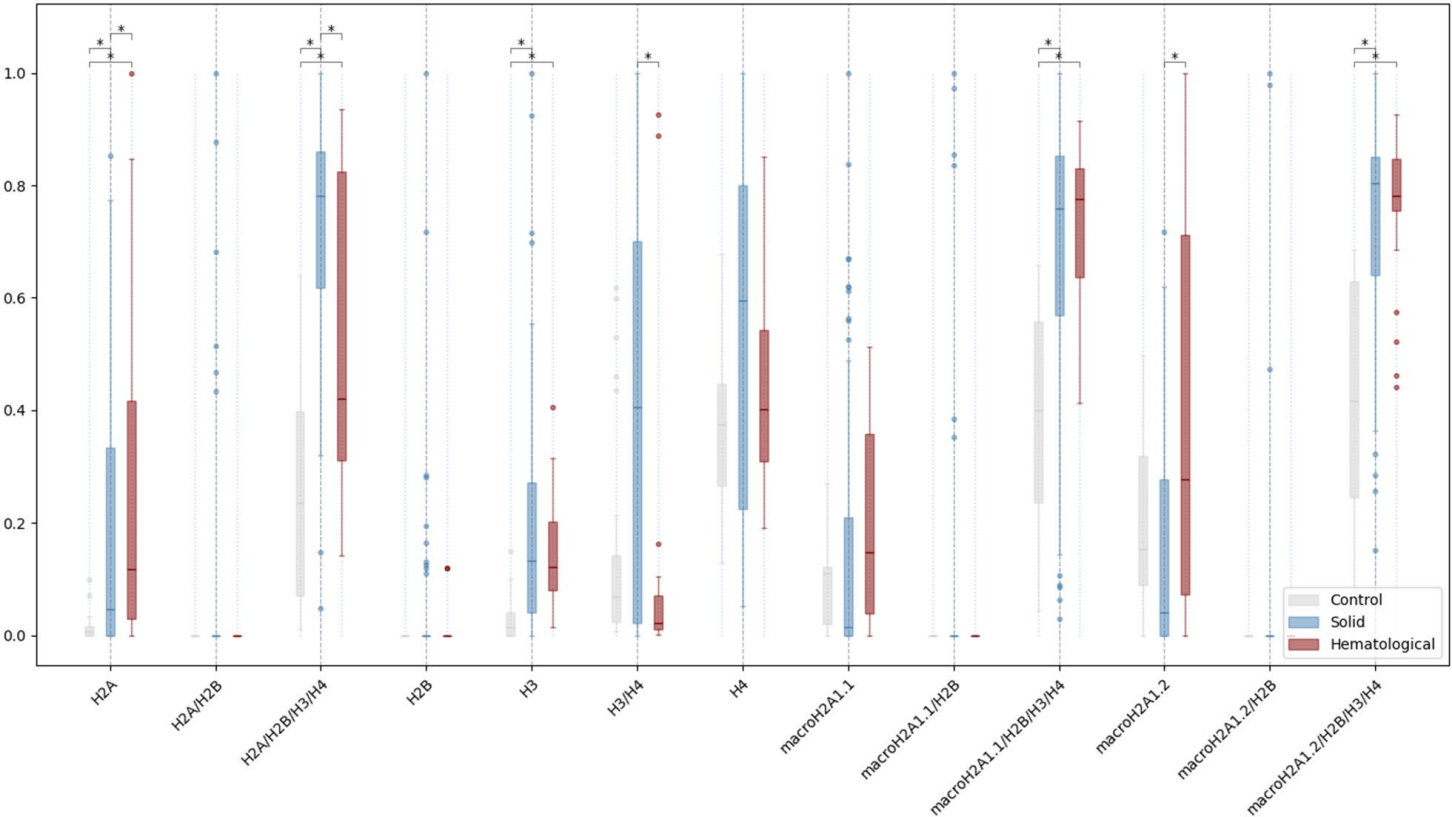
Relative abundance of circulating histones and histone complexes measured by ImageStreamX in healthy individuals (*n* = 30), patients with MDS (*n* = 43) or patients with cancer of solid origin (BC, NSCLC, SCLC, or CRC; total number of solid cancer patients = 74). Significance is indicated by asterisks: < 0.05 (*), < 0.01 (**), and < 0.001 (***) based on Kruskal–Wallis test followed by a post-hoc test

Next, to identify individual differences in plasma histone profiles across the populations, the abundance of each of the analyzed histones, histone dimers, and nucleosomes was compared. ImageStream(X) analysis uncovered and significant increases in the abundance of the following histones in the blood of patients with solid malignancies (grouping the results for CRC, SCLC, NSCLC, and BC) or MDS *versus* healthy controls: H2A (*p* < 0.01), H2A/H2B/H3/H4 (*p* < 0.01), H3 (*p* < 0.01), macroH2A1.1/H2B/H3/H4 (*p* < 0.01), and macroH2A1.2/H2B/H3/H4 (*p* < 0.01) (Fig. 2). Interestingly, a significantly increased abundance of histones H2A and macroH2A1.2 (*p* < 0.01) and a decreased abundance of H2A/H2B/H3/H4 and H3/H4 histone complexes (*p* < 0.01) specifically discriminated between MDS and solid cancers (Fig. 2). No differences between the three groups (controls, solid cancers, blood cancers) were detected regarding the expression macroH2A1.1, while H2A/H2B dimer, H2B, H4, macroH2A1.1/H2B, and macroH2A1.2/H2B dimers were detectable only in a few patients (Fig. 2).

**Fig. 3:**
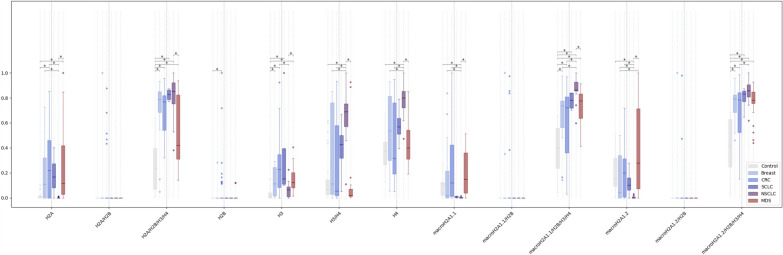
Relative abundance of circulating histones and histone complexes measured by ImageStreamX in healthy individuals (n = 30), patients with MDS (n = 43) or patients with cancer of solid origin: BC (*n* = 16), NSCLC (*n* = 15), SCLC (*n* = 4), or CRC (*n* = 39). Significance is indicated by asterisks: < 0.05 (*), < 0.01 (**), and < 0.001 (***) based on Kruskal–Wallis test followed by a post-hoc test

Subsequently, we plotted the same data according to individual types of solid tumors (CRC, SCLC, NSCLC, and BC) (Fig. 3). Several significant differences were observed in the levels of individual histone species between discrete solid tumor entities; remarkably, NSCLC patients displayed the highest circulating levels of H2A/H2B/H3/H4, H3/H4, H4, macro H2A1.1/H2B/H3/H4, and macroH2A1.2/H2B/H3/H4 (*p* < 0.01), and the lowest levels of single histones H2A, H3, macroH2A1.1, and macroH2A1.2 (*p* < 0.01) compared to CRC, SCLS and BC (Fig. 3). Consistent with the previous plotting comparing solid cancers *versus* MDS (Fig. 2), H2A/H2B dimer, H2B, macroH2A1.1/H2B, and macroH2A1.2/H2B dimers were detectable only in a few patients affected by the single solid cancer types (Fig. 3). Overall, these findings offer a proof-of-concept of a new histone-based liquid biopsy for the rapid detection of solid *versus* blood-borne malignancies, which is differentially associated with aging and may enable tumor epigenetic characterization by minimally invasive means.

### Circulating histones correlate with clinical parameters in solid malignancies and MDS

**Fig. 4:**
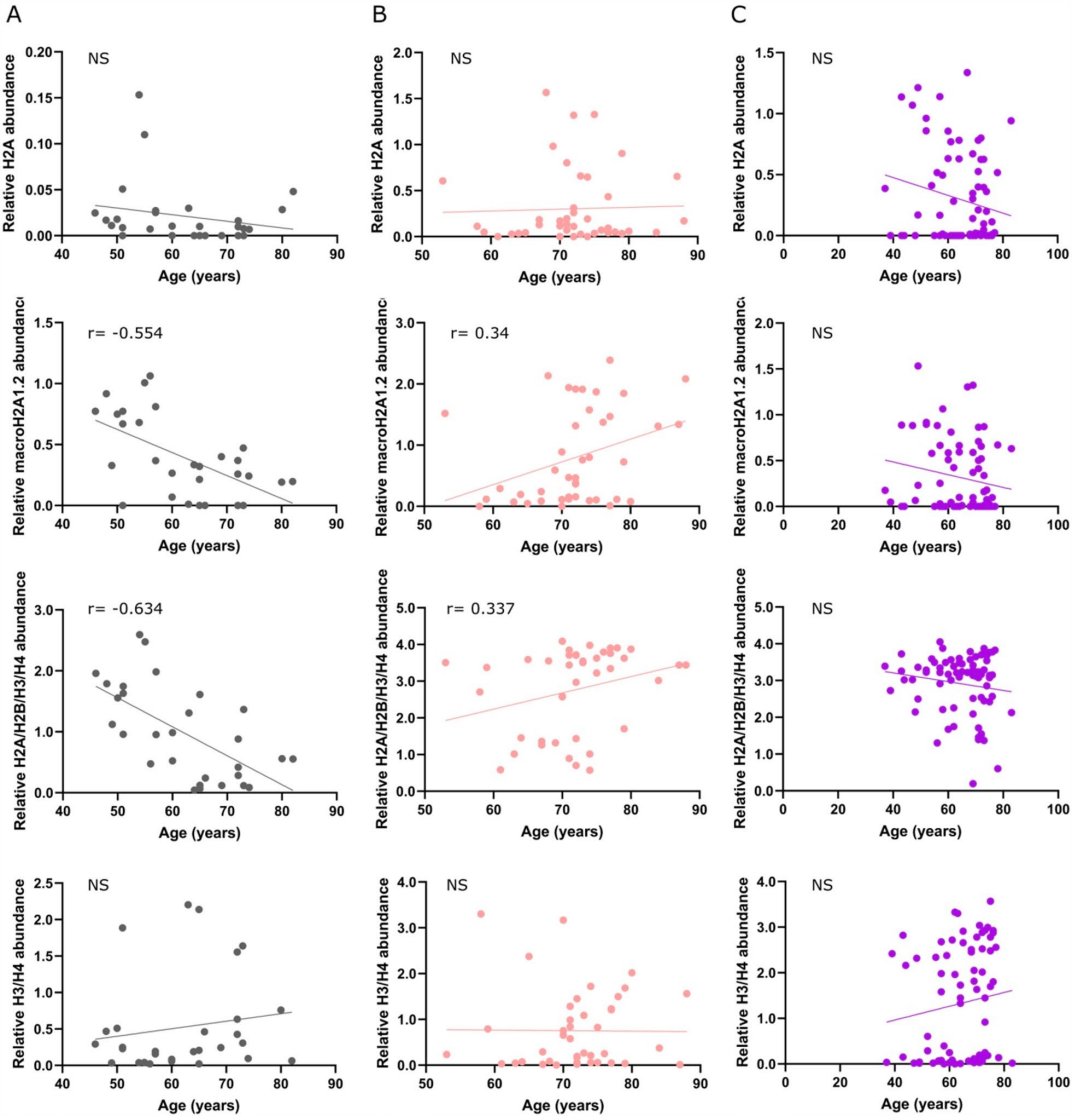
Correlation of H2A, macroH2A1.2, H2A/H2B/H3/H4, or H3/H4 and age in **A** healthy people (gray), **B** MDS patients (pink), and **C** patients with cancer of solid origin (purple). **A**-**C** Each symbol in the graphs represents an independent sample. The correlation coefficient (r) is shown on each of the graphs

The tissue/cellular origin of these circulating histones in the blood of cancer patients is unknown. Within tissues, histones are aging markers, and their levels generally decrease with organismal aging in mammals [51, 52]. Such a relationship has never been studied with circulating histones in humans. We therefore plotted the levels of the histone species differentially expressed between solid cancers and MDS (H2A, macroH2A1.2, H2A/H2B/H3/H4, and H3/H4) against the age of the patients; results are shown in Fig. 3. Interestingly, in healthy subjects, a moderate negative linear correlation was detected between the levels of macroH2A1.2 (*r* = − 0.5541) or H2A/H2B/H3/H4 (*r* = − 0.6337) and age (Fig. 4A). On the contrary, in MDS patients, a weak positive linear correlation was present between the levels of macroH2A1.2 (*r* = − 0.3397) or H2A/H2B/H3/H4 (r = − 0.3370) and age (Fig. 4B). No correlation between circulating histone species levels and age was found in solid cancers (Fig. 4C).

To further understand the potential value of circulating histones in clinical practice for MDS, we correlated their abundance levels with disease parameters. Most of the histones and histone complexes negatively correlated with hemoglobin (Hb) and percentage of blasts in the bone marrow (Supplementary Table 1). Specific plasma histone profile was not associated with death at the time of analysis (Supplementary Fig. 2A). No significant differences in plasma histones were observed following patient stratification based on cellularity (Supplementary Fig. 2B) or Revised International Prognostic Scoring System risk (IPSS-R) score (Supplementary Fig. 2C).

## Discussion

The goal of this study was to compare the levels of individual histones and histone complexes between healthy individuals, patients with MDS, and solid cancer patients (CRC, NSCLC, SCLC BC), using imaging flow cytometry that measures quantitatively their relative abundance. The latter approach, compared to ELISA—measuring total abundance, is faster and more multiplex, while preserving a high sensitivity and displaying the visual power of microscopy to provide extensive information on particles of interest, notably histones [25]. Several studies have demonstrated extracellular histones H3 and/or H4 as potential and nonspecific mediators of lethal systemic inflammatory diseases, but circulating histones are understudied as LB in cancer. The most widely studied LB, cfDNA, has proven a valuable cancer-characterization method in both cancers of solid tumor or hematological origin [29, 53, 54]. Nevertheless, the short half-life of cfDNA in circulation could limit its sensitivity [55, 56], especially in the cases of low-abundance cfDNA fragments. On the other hand, histone proteins have a long half-life and are stable in the blood. In terms of the diagnostic value of LB, anti-nucleosome antibodies have been shown to be > twofold more sensitive compared to anti-DNA antibodies in the detection of autoimmune diseases [57]. Recently, there have been tremendous advances in cancer early detection methods, including tissue analysis, urinalysis, and tumor markers. However, besides hypothesis-driven cancer-specific cfDNA, which reflects mutational profiles, LB/biomarker signatures that are easy to detect, unbiased, fast, non-invasive, and able to discriminate between hematological and solid cancers are currently lacking. In a cohort of 555 naïve cancer patients, including indiscriminately solid cancers (lung, breast, and gastrointestinal) and MDS, circulating levels of cardio-oncological marker Growth Differentiation Factor 15 (GDF-15, measured by ELISA) were associated with all-cause mortality in the total cohort, whereas in the subgroup analysis, association with outcome remained significant only for solid tumors but not for MDS [58]. Other studies have compared healthy individuals to patients with MDS or solid tumors by analyzing circulating histone signatures. However, in most cases, the circulating histones are analyzed on the level of nucleosomes or post-translational modifications (PTMs) in both hematological and solid malignancies [25, 59, 60]. For instance, elevated levels of plasma H3.1 nucleosomes and eight histone PTMs were reported in patients with non-Hodgkin lymphoma (NHL) compared to healthy controls. Subsequent analyses have indicated the potential value of these PTMs for monitoring disease progression and/or treatment response [61]. In contrast, our study focused on differential levels of structural components of the nucleosomes, including individual histones and histone variants, dimers, and nucleosomes with core H2A histone protein or H2A variants. Previous work demonstrated that circulating Nu.Q-H3.1 nucleosome levels are elevated in patients with hematological cancers and in patients with solid cancers, compared to healthy controls. Interestingly, patients with hematological cancers showed elevated levels of H3.1 nucleosomes, compared to solid cancer patients [59]. In contrast, we found decreased levels of plasma H2A-nucleosomes in MDS vs. solid cancers and comparable levels of plasma macroH2A1.1 nucleosomes and macroH2A1.2-nucleosomes between the two cohorts, indicating the importance of histone variants in biomarker research. Our future studies aim to address the circulating histone profile in distinct hematological malignancies in a longitudinal manner. Undoubtedly, the differences we identified cannot be regarded as a differential diagnostic test, as the need to distinguish between such clinically distinct diseases is relatively rare, and highly specific diagnostic tools are routinely available when such differentiation is required. However, it should be noted that myelodysplastic neoplasms may occasionally present with extramedullary manifestations, including cases without bone marrow involvement, with or without distant metastases, thus mimicking various solid tumors. This has been reported even for tumor types included in the present study: BC [62–64], LC [65], andCRC [66]. The diagnosis of such cases is always challenging. An open question for future research is to what extent the molecular or circulating biomarker profile of each tumor type remains stable or undergoes changes in the context of atypical localization or the development of secondary malignancies, e.g., secondary MDS, and whether such investigations may contribute to comprehensive diagnostics. Rather than replacing existing diagnostic methods, we aimed to identify one or more markers that could improve diagnostic accuracy, expedite patient triage, and support cancer subtyping or characterization. In future studies, we plan to compare the value of circulating histones with established circulating biomarkers in cancer research. Their integration with other liquid biopsy methods will be essential for adoption into routine clinical practice. Another key objective is to evaluate circulating histone profiles in a longitudinal setting to assess their potential for screening and disease monitoring.

Here, while we found a general increase in most circulating histone species in cancer (MDS or solid) compared to healthy patients, we identified for the first time a circulating histone signature able to discriminate a hematological malignancy *versus* solid malignancies, consisting in an increased abundance of histones H2A and its variant macroH2A1.2 and a decreased abundance of H2A/H2B/H3/H4 and H3/H4 histone complexes. At the epigenetic level, aging is associated with an increased distance between nucleosomes, which can be used to conduct predictions and classifications on a person’s age [67]. Interestingly, although most of our MDS and solid cancer patients were diagnosed and enrolled in the study between their 6th and 8th decade of life, opposite correlations were found between macroH2A1.2 and H2A/H2B/H3/H4 circulating levels and age in healthy controls *versus* MDS, which may not only reflect absolute differences but could point to different mechanisms of epigenetic accelerated aging [68]. In this respect, as we have shown earlier, histone macroH2A1, in particular, participates in the formation of senescent cells [40, 69] and is a key player in the cellular and molecular features of MDS [46]. Selective degradation of histone species in the blood could theoretically account, at least in part, for their differential abundance within cancer types, but this has not been documented yet. A limitation of the present study is that it does not analyze the ~ 100 known PTMs, some of which can be deregulated in cancers [70, 71]. PTMs, such as acetylation, methylation, and phosphorylation, can affect histone–histone interactions within the nucleosome, and the binding of proteins that influence chromatin structure and function [72]. PTMs can thus impact nucleosome stability, making it more or less likely to disassemble, which could potentially reflect on the relative abundance of circulating histones in cancer patients. Moreover, our study does not provide information about the tissue/cellular origin of the circulating histones in the blood of cancer patients. Advanced nucleosome footprint studies have identified the cfDNA signature of hematopoietic (in particular myeloid and lymphoid) lineages, which are the main contributors to cfDNA in healthy individuals, with additional small contributions from non-hematopoietic tissues in individuals with solid cancers [73–75]. Assuming that both cfDNA and histones are released in the bloodstream upon cell death in the hematopoietic compartment, we hypothesize that the differences in circulating histone levels observed between solid cancers and MDS may be at least in part connected to the disorder of hematopoietic cell production inherent to MDS. MDS is reportedly more prevalent in males; stratification of our cohort based on sex was not performed due to the relatively small sample size, although a weak positive correlation was observed between most circulating histones and age (Supplementary Table 1). The profile of circulating histone in the plasma of NSCLC patients was markedly different from SCLS or other cancer patients, in particular displaying lower levels of H2A, H3, and macroH2A1.2 (Fig. 3). In this respect, it has long been reported that, compared to SCLC cells, NSCLC cells do not fully execute apoptosis, as the apoptotic process in NSCLC cells seems to be blocked downstream of caspase activation [76, 77], which may explain decreased plasma histone levels.

## Conclusions

In conclusion, in this study, we found that one type of hematological malignancy, MDS, and solid cancers could be discriminated among themselves for an increased abundance of plasma histones H2A and macroH2A1.2, and a decreased abundance of H2A/H2B/H3/H4 and H3/H4 histone complexes. These findings provide valuable insights into cancer diagnosis, paving the way for novel screening strategies. ImageStream(X), used in this study, is the first commercially available imaging flow cytometer, and it is a large and expensive instrument. Technologies to miniaturize imaging flow cytometers, combined with artificial intelligence approaches, could contribute to developing more effective cancer screening methods by incorporating new time-sensitive and cheaper point-of-care diagnostic devices. In turn, these devices could favor an early triage and categorize patients with generic cancer symptoms.

## Supplementary Information

Supplementary Material 1

Supplementary Material 2

Supplementary Material 3

## Data Availability

The data that support the findings of this study and the code/scripts used for their analysis are available from the corresponding author upon reasonable request.

## Abbreviations

ALC: Absolute lymphocyte count
AML: Acute myeloid leukemia
ANC: Absolute neutrophils count
BC: Breast cancer
B2-MG: Beta-2 microglobulin
cfDNA: Cell-free DNA
CRC: ColoRectal cancer
CTCs: Circulating tumor cells
DIPG: Diffuse intrinsic pontine glioma
EPO: Erythropoietin
EVs: Extracellular vesicles
Hb: Hemoglobin
LDH: Lactate dehydrogenase
MDS: Myelodysplastic syndrome
Plt: Platelets
NSCLC: Non-small cell lung cancer
SCLC: Small cell lung cancer
WBC: White blood cells
