# Supplementary material for: Histone-based liquid biopsy discriminates between myelodysplastic syndrome and solid malignancies
Source: Clin Epigenetics. 2025 Nov 21;17:199. doi: 10.1186/s13148-025-01995-w (PMC12639983; doi:10.1186/s13148-025-01995-w)
Supplement: Supplementary file 1 — Supplementary Material 1 [file 13148_2025_1995_MOESM1_ESM.docx]

**Supplementary Table 1.** Correlation coefficients (r) of plasma levels of histones and histone complexes and clinical characteristics of MDS patients.
