# Supplementary figures and images for: Histone-based liquid biopsy discriminates between myelodysplastic syndrome and solid malignancies
Source: Clin Epigenetics. 2025 Nov 21;17:199. doi: 10.1186/s13148-025-01995-w (PMC12639983; doi:10.1186/s13148-025-01995-w)

A

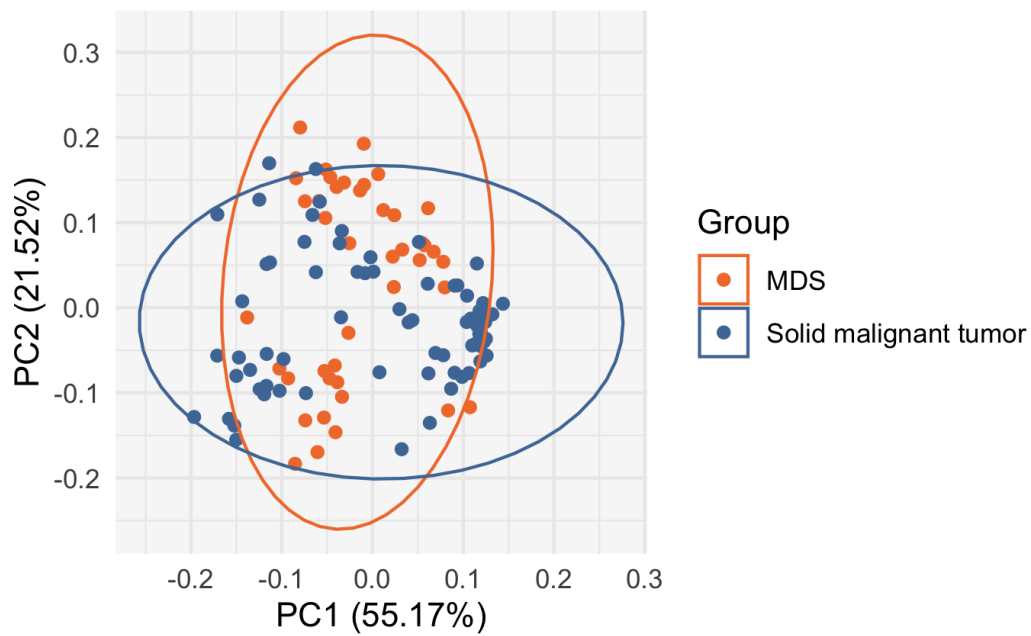

B

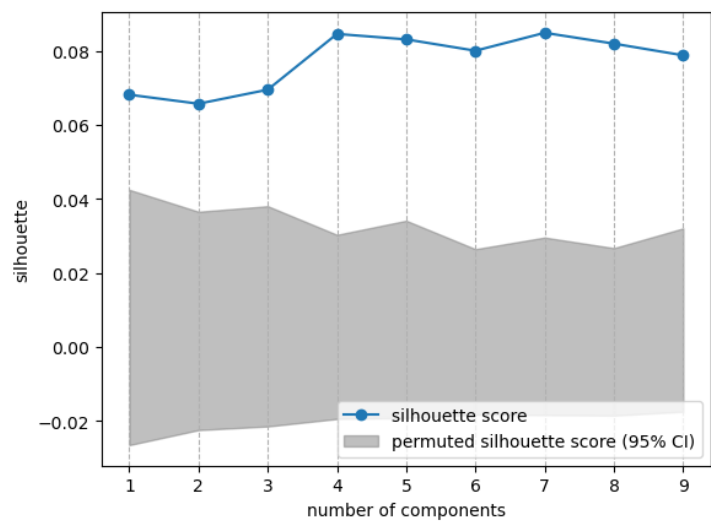

C

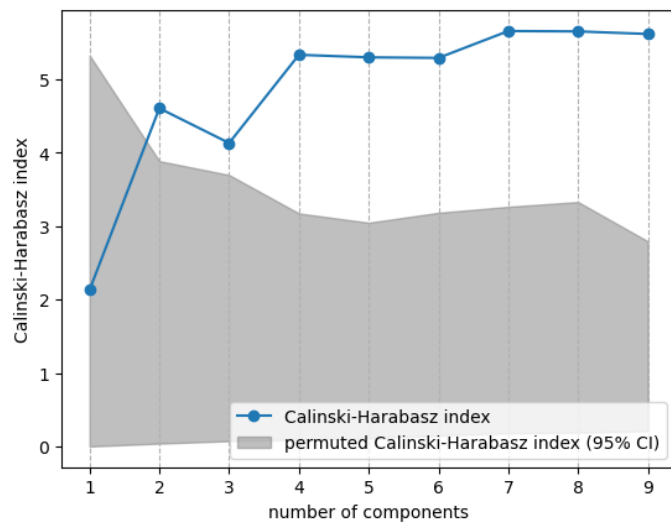

Supplement: Supplementary file 2 — Supplementary Material 2 [file 13148_2025_1995_MOESM2_ESM.pdf]

**A**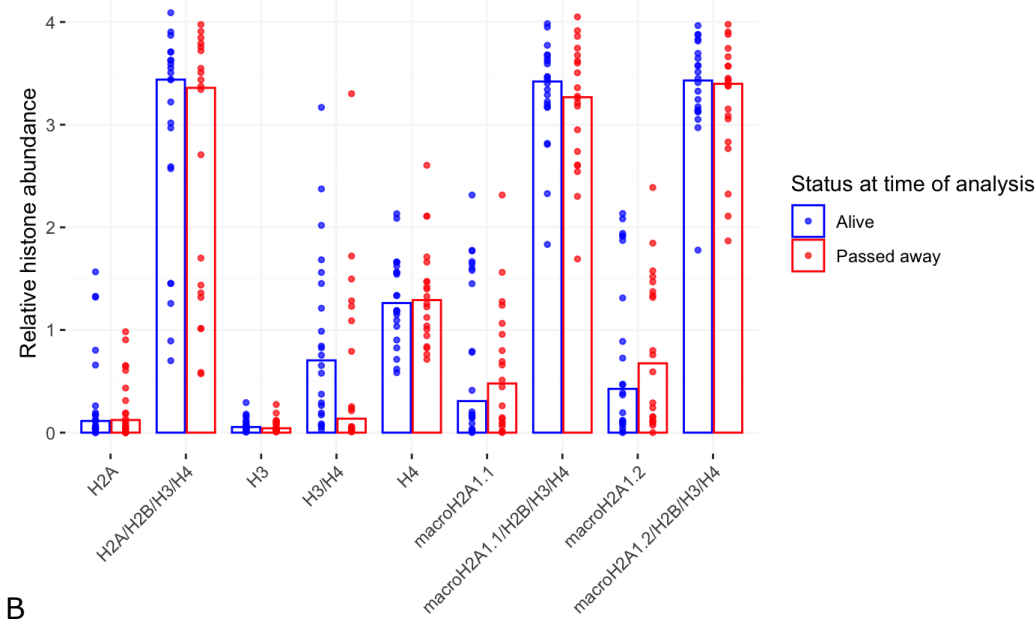**B**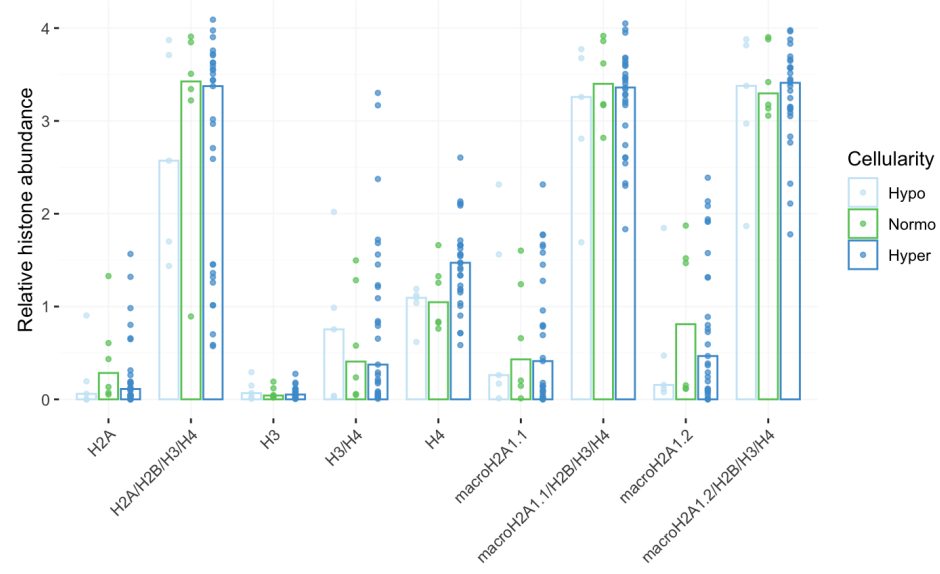**C**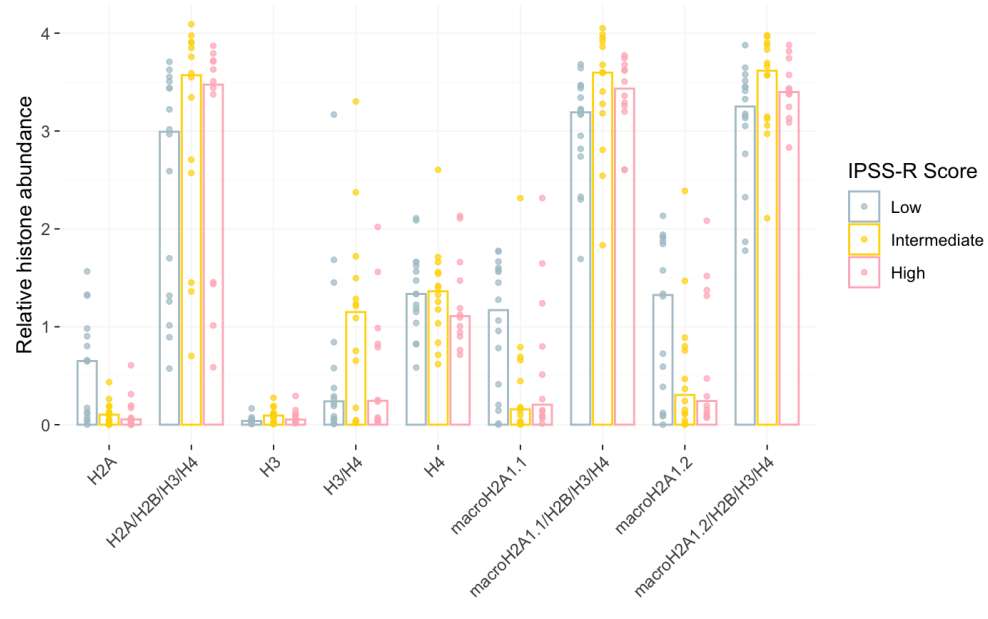

Supplement: Supplementary file 3 — Supplementary Material 3 [file 13148_2025_1995_MOESM3_ESM.pdf]
